# Stromal striae: a new insight into corneal physiology and mechanics

**DOI:** 10.1038/s41598-017-13194-6

**Published:** 2017-10-19

**Authors:** Kate Grieve, Djida Ghoubay, Cristina Georgeon, Gael Latour, Amir Nahas, Karsten Plamann, Caroline Crotti, Romain Bocheux, Marie Borderie, Thu-Mai Nguyen, Felipe Andreiuolo, Marie-Claire Schanne-Klein, Vincent Borderie

**Affiliations:** 1 CHNO des Quinze Vingts, INSERM-DHOS CIC 503, Paris, France; 2 Institut de la Vision, Sorbonne Universités, UPMC Univ Paris 06, INSERM, CNRS, Paris, France; 3 Laboratoire Imagerie et Modélisation en Neurobiologie et Cancérologie, Univ. Paris-Sud, CNRS, Université Paris-Saclay, Orsay, France; 4 Institut Langevin, Paris, France; 5 ENSTA ParisTech, Ecole polytechnique, CNRS, Université Paris-Saclay, Palaiseau, France; 6 Laboratoire d’Optique et Biosciences, Ecole polytechnique, CNRS, INSERM U1182,Université Paris-Saclay, Palaiseau, France

## Abstract

We uncover the significance of a previously unappreciated structural feature in corneal stroma, important to its biomechanics. Vogt striae are a known clinical indicator of keratoconus, and consist of dark, vertical lines crossing the corneal depth. However we detected stromal striae in most corneas, not only keratoconus. We observed striae with multiple imaging modalities in 82% of 118 human corneas, with pathology-specific differences. Striae generally depart from anchor points at Descemet’s membrane in the posterior stroma obliquely in a V-shape, whereas in keratoconus, striae depart vertically from posterior toward anterior stroma. Optical coherence tomography shear wave elastography showed discontinuity of rigidity, and second harmonic generation and scanning electron microscopies showed undulation of lamellae at striae locations. Striae visibility decreased beyond physiological pressure and increased beyond physiological hydration. Immunohistology revealed striae to predominantly contain collagen VI, lumican and keratocan. The role of these regions of collagen VI linking sets of lamellae may be to absorb increases in intraocular pressure and external shocks.

## Introduction

Stromal striae, also termed Vogt striae or banding patterns, are frequently used as an indicator of keratoconus^1–6^. Viewed using a slit-lamp, their appearance is described as a series of vertical striae running from posterior to anterior stroma^1,2^, becoming more visible and more frequent with advancing keratoconus. Their appearance is similar in optical coherence tomography (OCT) cross-sections^3^. Occasionally, horizontal striae are also observed by slit-lamp^4^. Recently, *in vivo* confocal microscopy (CM) has emerged as an imaging modality capable of providing microscopic en face views of the corneal *in vivo*. In CM en face views, Vogt striae are described as criss-crossing striae in posterior stroma of keratoconic corneas^5–7^.

Full-field OCM is a variant on the OCT principle^8^ that provides microscopic en face and cross-sectional views using white light and microscope objectives to increase resolution to 1 µm in three-dimensions^9,10^. It has previously been used in corneal imaging^11–15^. In a recent study, the appearance of Vogt striae was assessed in keratoconic corneas using FFOCM, and was found to be similar to that described using OCT, CM and slit-lamp^16^. However these striae were also observed in non-keratoconic corneas, including both pathological and normal samples. Based on these observations, we studied a series of images captured with OCT, CM, FFOCM, and histology to search for striae in a series of normal, keratoconic and non-keratoconic pathological corneas. The aim of the current study is firstly to describe the striae patterns found with each modality and in each syndrome of pathology, in order to discover which aspects of the appearance of striae are specific to keratoconus, and whether other aspects could indicate other pathologies. We also address the question of what they may signify in terms of corneal microstructure and mechanics. To explore the corneal microstructure, corneas were immunolabeled for collagen types, and sliced with electron microscopy to look at the micro and nano scales; corneas were also imaged with second harmonic generation microscopy (SHG) to visualize collagen, including polarization-resolved SHG to map orientation of collagen lamellae in transverse sections^17^. To investigate mechanics, optical coherence tomography shear wave elastography (OCT-SWE), a recently developed technique that provides stiffness maps with a 20 µm resolution, was able to reveal discontinuities in stiffness of the observed striae in relation to the surrounding tissue. OCT-SWE, SHG and CM monitored band visibility in relation to changing intraocular pressure (IOP).

We postulate that striae are associated with the biomechanical properties of the cornea and that they represent zones of structural importance to the maintenance of corneal shape and biomechanical properties that may be affected by pathology.

## Results

Histograms presenting this section’s data in more detail are available in Fig. sup 1.1.

**Figure 1:**
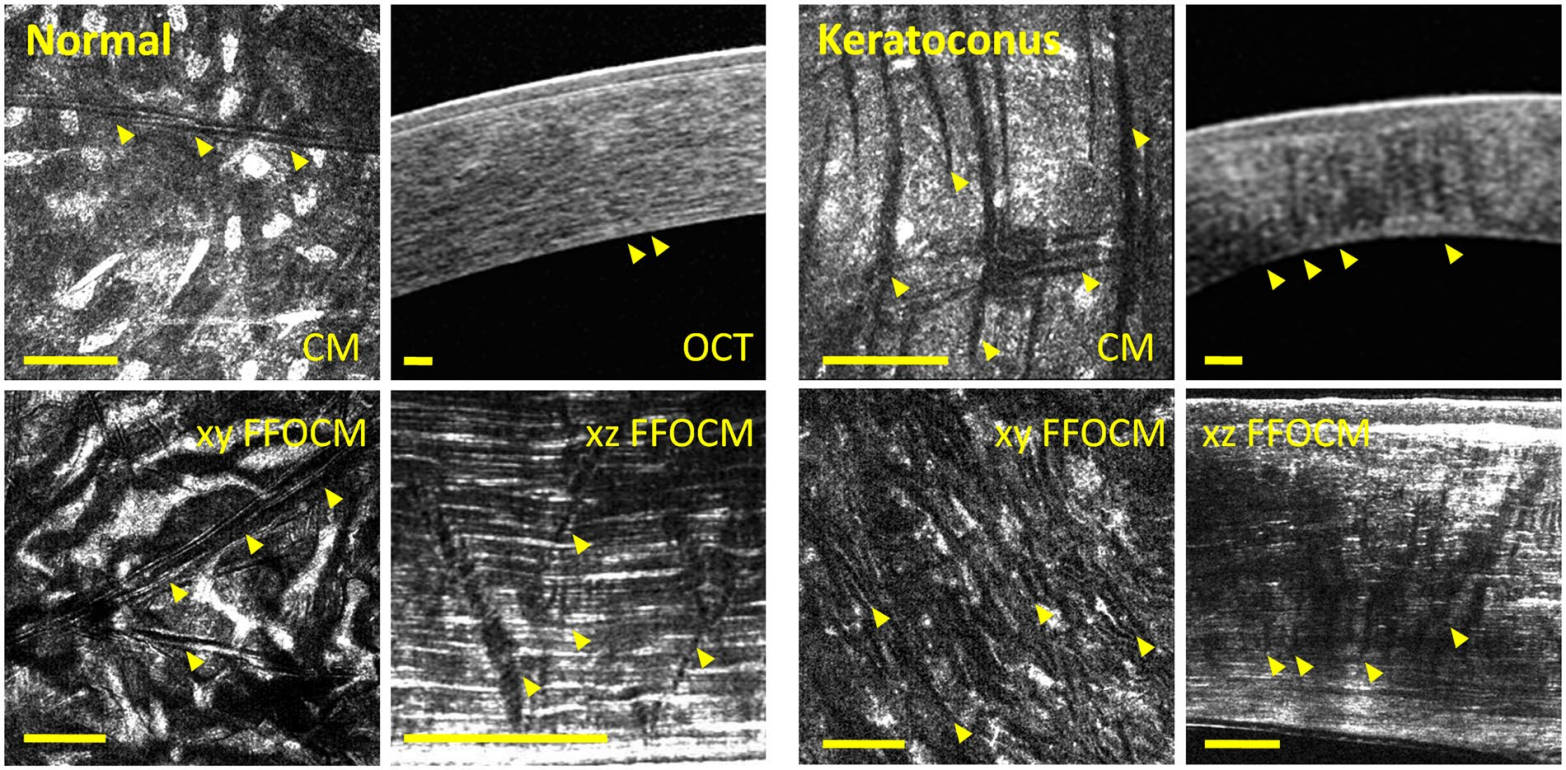
Striae in normal (left panels) versus keratoconus (right panels) cornea. Viewed with (clockwise) confocal microscopy (CM), OCT, FFOCM cross-sectional xz and en face xy views. Arrowheads indicate striae. Striae in normal cornea were few and oblique, while striae in keratoconus were numerous and vertical. Scale bars show 100 µm.

**Figure 2:**
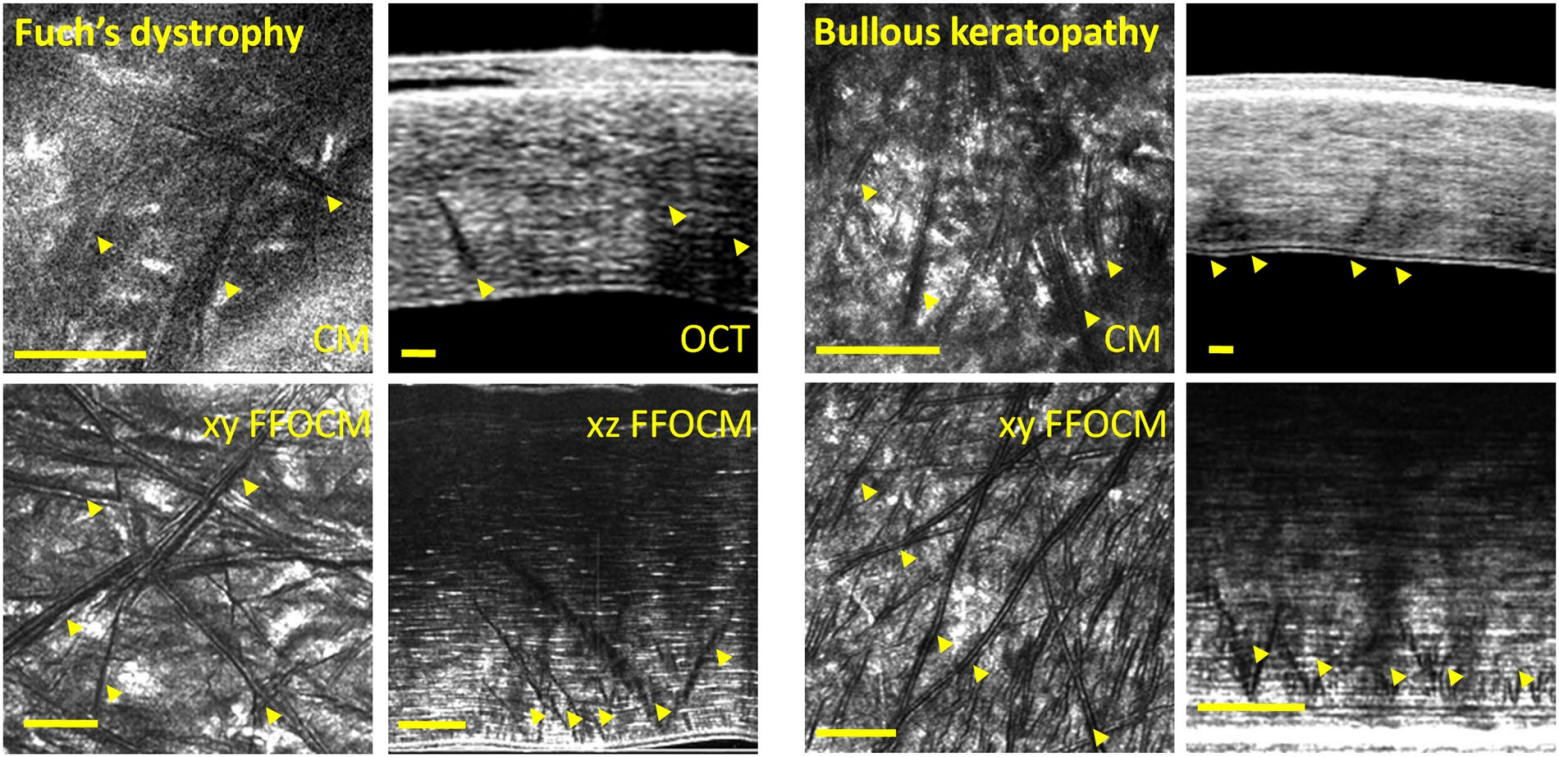
Striae in corneas with edema: Fuch’s dystrophy (left panels), bullous keratopathy (right panels). Viewed with (clockwise) confocal microscopy (CM), OCT, FFOCM cross-sectional xz and en face xy views. Arrowheads indicate striae. Striae in edematous corneas were few and oblique. Scale bars show 100 µm.

Striae were observed in 82% of the corneas we observed (n = 118) with at least one imaging modality (histology 74% (n = 23), CM 64% (n = 107), FFOCM 67% (n = 55) and SD-OCT 38% (n = 101), Fig. sup 1.1). In cross-sectional views, striae depart from anchor points at Descemet’s membrane in the posterior stroma (Figs 1 and 2). They cross the corneal stroma toward Bowman’s layer. They present as dark striae, with an overall mean of 6 striae per mm, of width 20 µm, and length covering 42% of the stromal thickness. In the plane parallel to the ocular surface, striae often criss-cross over each other as seen in CM, FFOCM and histology en face images (Figs 1 and 2). Striae number and width diminished with age (p = 0.008 and p = 0.03 respectively).

### Morphology and structure of stromal striae in normal cornea

Striae in normal cornea were few, short and oblique (Fig. 1). This was also the case in other mammalian species (i.e. mouse, macaque, Fig. sup 1.2).

**Figure 3:**
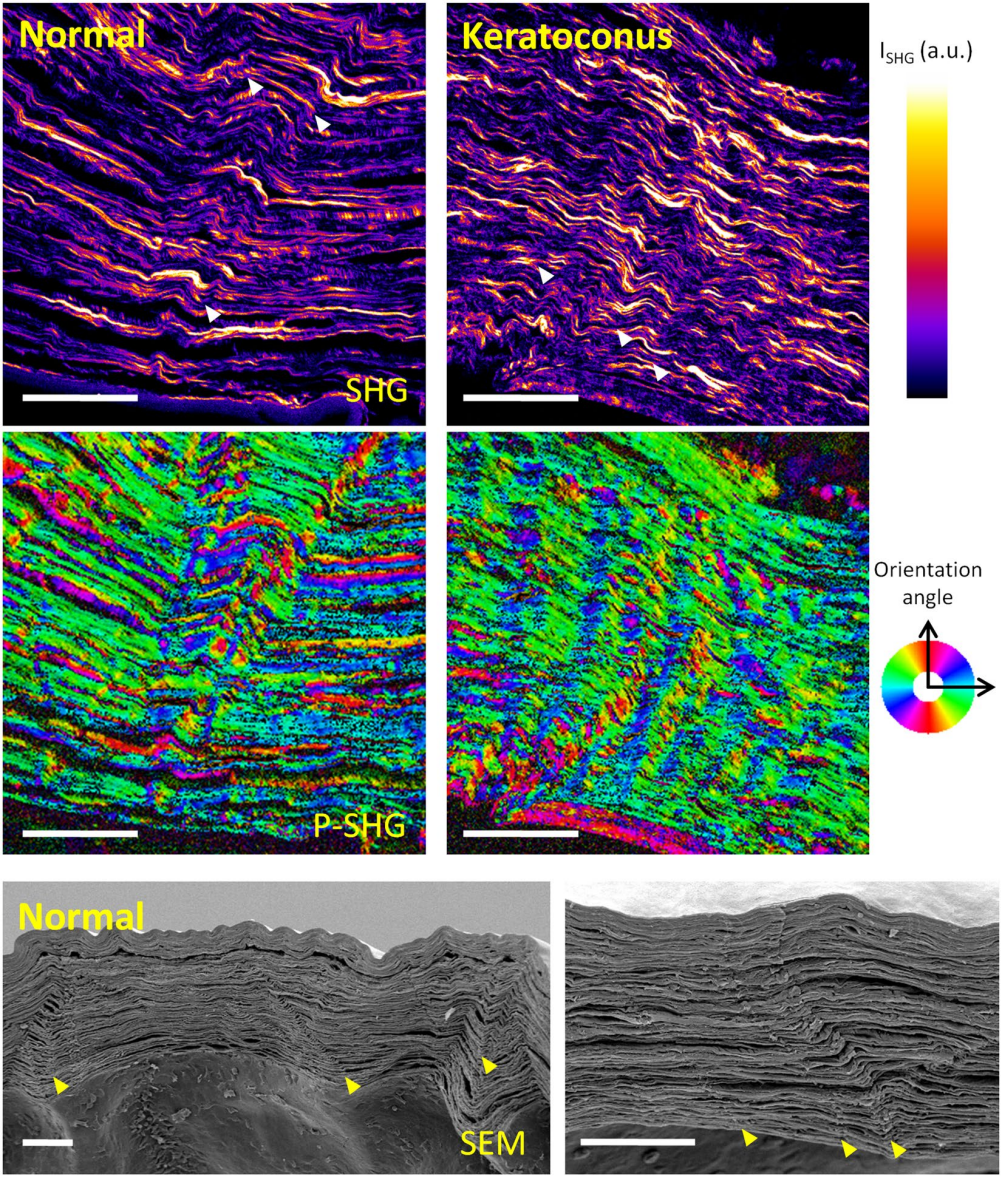
Striae visualized by SHG (top) and P-SHG (center) in normal (left) and keratoconic (right) and in normal cornea with scanning electron microscopy, SEM (bottom). Human donor corneas. Top: SHG intensity, center: orientation of collagen fibrils obtained from P-SHG. Look-up-tables are shown on the right side. Striae are more numerous in keratoconus. P-SHG orientation mapping shows that they correspond to undulations in continuous lamellae rather than ruptures. Scale bars show 50 µm. SEM (bottom) confirms that striae are undulations rather than ruptures in increasing zoom from left to right panels, where arrowheads highlight striae. Scale bars show 100 µm.

SHG microscopy (Fig. 3) showed that the striae correspond to undulations in continuous lamellae rather than ruptures. Polarization resolved data confirmed the change in the orientation on the fibril scale. The angular change was 72° +/− 13° on average (n = 18 measures in 2 corneas). Scanning electron microscopy confirmed the presence of criss-crossing lines in normal cornea corresponding on the micro and nanometric scales to undulations, as opposed to ruptures, in the regular arrangement of lamellae (Fig. 3).

We could demonstrate that striae did not correspond to folding artifacts due to the histological process using 3D SHG image stacks. We acquired a stack of images in micron depth steps through an artifactual fold in a histological slice, and were able to see the 3D form of the fold in SHG, while imaging a striation with the same technique shows that the striation is continuous with the collagen lamellae and integrated in the same 2D planes (Fig. sup 1.3).

Corneal hydration, monitored by imaging a human donor cornea using SD-OCT and CM during 3 days deturgescence (Fig. sup 1.4), indicated that striae are present at all hydration levels, and more visible when stromal hydration increases. Efforts were therefore made to record all data on non-edematous tissue.

**Figure 4:**
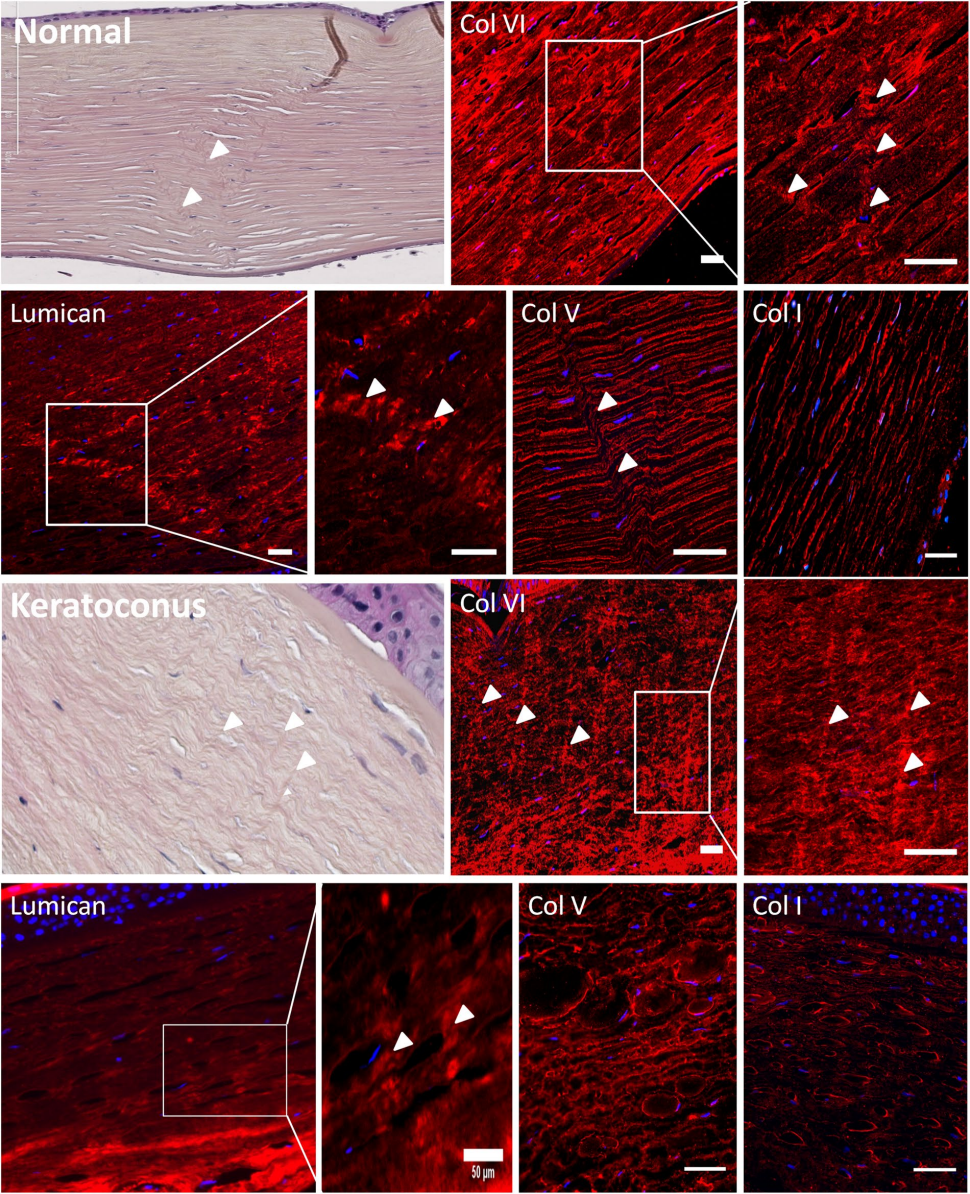
Labeling different collagen types in normal human donor, and keratoconus cornea. Striae were visible in collagen VI, lumican and keratocan (not shown). Stromal collagen fibrils that are made of collagen I and collagen V appear not to be involved in striae formation. Collagen VI presents a filamentous structure and it is involved in maintaining the interfibril space. Its density is increased at the level of striae. Arrowheads indicate striae. Scale bars show 50 µm.

Immunofluorescence revealed that striae were composed of collagen VI, lumican and keratocan and not of collagen I, IV or V (Fig. 4), in contrast to the rest of the corneal stroma which is composed predominantly of collagen I. This was seen in cross section (Fig. 4) and en face views (Fig. sup 1.5) of human and macaque (Fig. sup 1.5).

### Effect of posterior pressure on stromal striae in normal cornea

**Figure 5:**
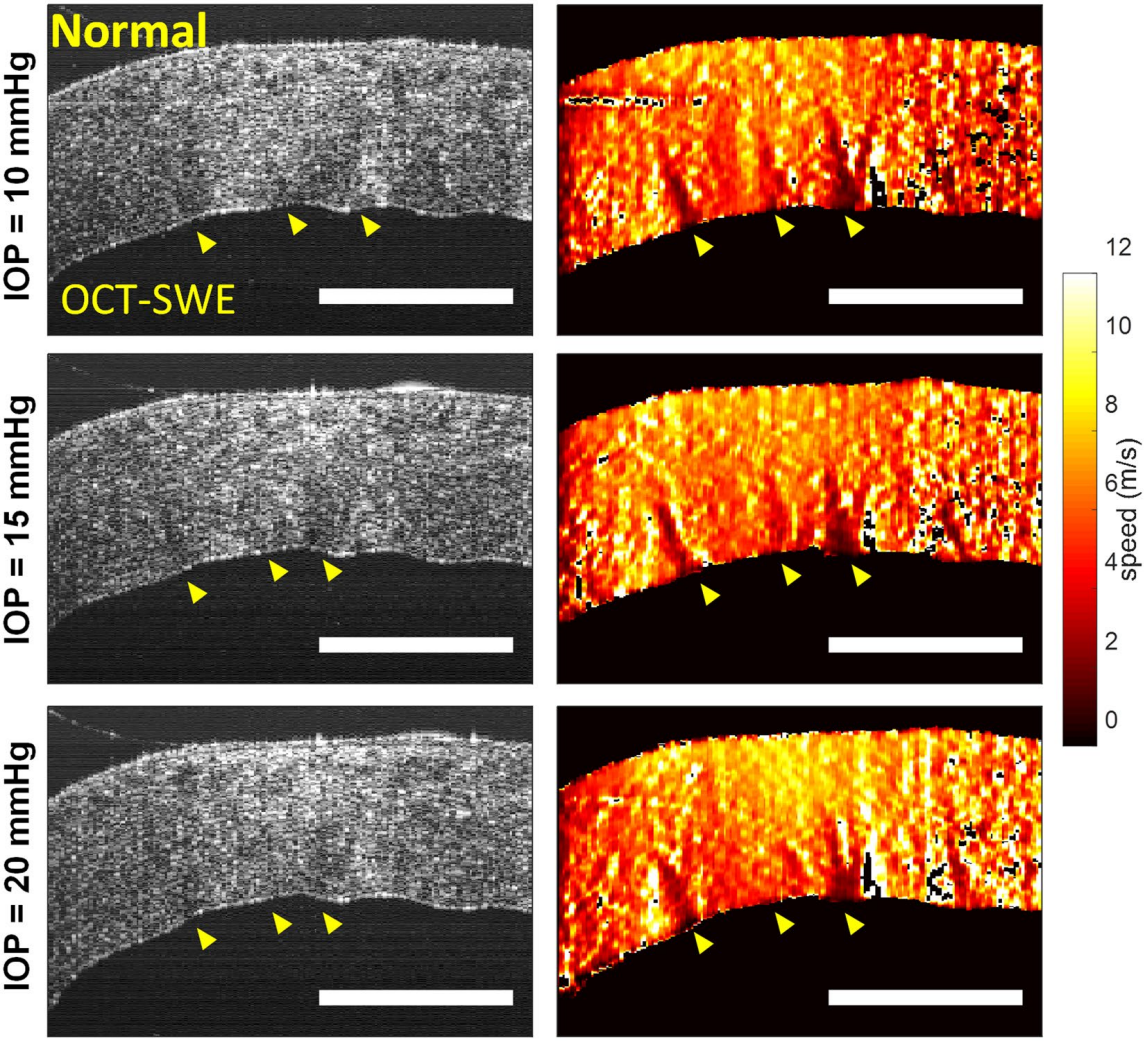
Optical coherence tomography (OCT) and shear wave elastography (SWE) images (left and right columns respectively) of a macaque cornea mounted in an artificial anterior chamber at different values of intraocular pressure (IOP). As indicated by the arrowheads, 3 striae are visible on the elastic maps (color scale: lower speed = softer region), while they are less obvious on the morphologic OCT image (gray scale) except for the far left stripe. The global propagation speed of the shear wave, from the right to the left hand side of the image, increases slightly with IOP, while contrast on striae reduces (see also Fig. 6). The rigidity maps show zones of discontinuity at striae, where shear wave speed cannot be accurately calculated due to rebounds, however global speed in striae zones reduces, indicating that striae are softer than surrounding stroma. Scale bars show 1mm.

**Figure 6:**
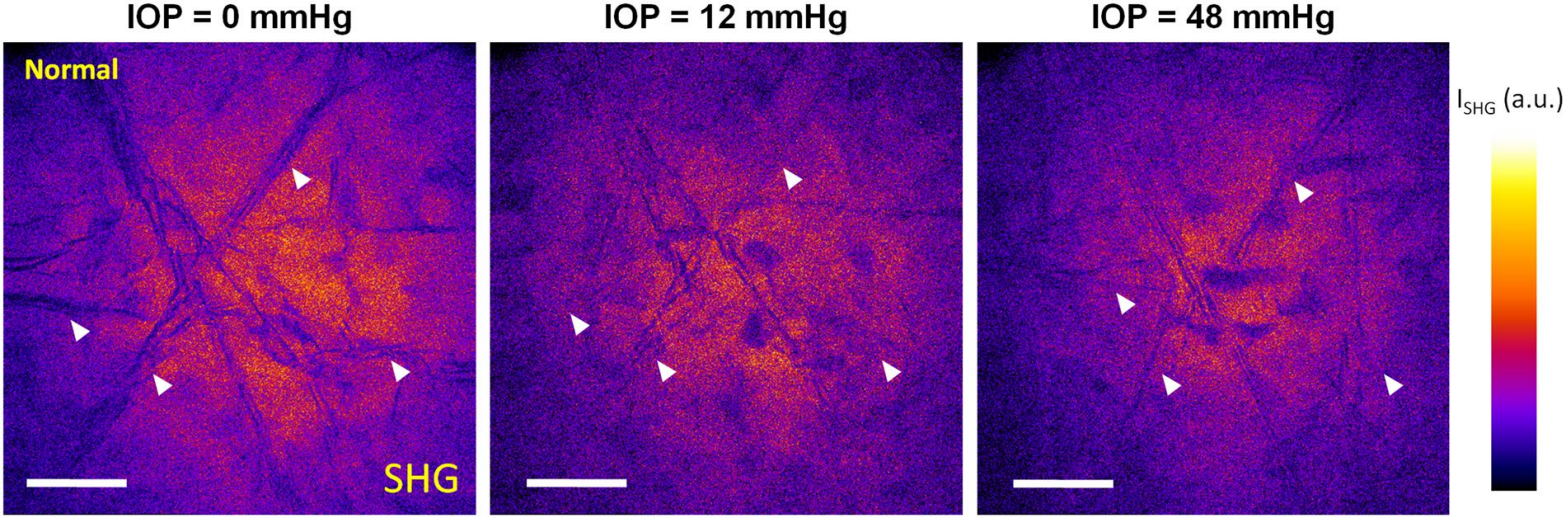
SHG imaging of a normal human donor cornea at increasing pressure. Striae exhibit a lower contrast at high pressure (48 mmHg) compared to physiological (12 mmHg) or zero pressure.

OCT-SWE revealed striae in normal donor and normal macaque cornea mounted in an artificial anterior chamber at different values of pressure, mimicking variations in the intraocular pressure (Fig. 5). The same experimental setting was used to assess striae at higher resolution with CM and SHG (Fig. 6), where the striae became less visible with increasing pressure. Striae exhibited a lower contrast at high pressure (48 mm/Hg) compared to physiological pressure in mid and posterior stroma. The contrast measured in SHG images decreased by a factor 1.05 +/− 0.40 in mid stroma and 1.35 +/− 0.38 in the posterior stroma (n = 6 corneas).

### Stromal striae in various corneal disorders

Differences in detection of striae according to syndrome were not statistically significant.

Striae in keratoconus were significantly more numerous than in normal (p = 0.01) or edematous (p = 0.01) cornea, and more vertical (p = 0.00001) than in corneas that were normal, edematous or contained opacities. Striae in corneas with opacities were significantly longer than all others (keratoconus p = 0.01, normal p = 0.001, edema p = 0.02). Differences in striae width according to syndrome were not significant.

Vertical striae in OCT and FFOCM cross-sections in keratoconus corresponded to parallel (not criss-crossing) lines in en face views in CM and FFOCM (Fig. 1). Oblique striae in cross-sections in other pathologies and normals corresponded to criss-crossing lines in en face views in CM and FFOCM (Figs 1 and 2).

In cases of keratoconus where an intrastromal ring segment had been inserted, striae were observed surrounding the segment in orientations that followed the segment form (Fig. sup 1.6). Intrastromal ring segments are rigid implants that aim to flatten the cornea in keratoconus patients, by pulling on the central cornea in a guy-rope fashion to flatten it. In cases of keratoconus where patients underwent cross-linking treatment, striae appearance remained constant while surrounding anterior stroma became more hyperreflective after cross-linking (Fig. sup 1.6). No significant differences in striae parameters (number, width, length, orientation) were detected when comparing before and after implant, or cross-linking, in central cornea.

## Discussion

### Prevalence of stromal striae

Striae were observed in 82% of the corneas we observed with at least one imaging modality. They were more rarely detected in OCT than in higher resolution modalities as their width is at the lateral resolution limit of the OCT system used. Striae are not just associated with keratoconus, although we postulate that they straighten to a vertical orientation aligned along the cone axis in keratoconus, due to the corneal conic shape. In single sections, they are difficult to differentiate from folds caused by slicing and inclusion artifacts in histology, and so may have been overlooked in the past. However the OCT, FFOCM and CM views demonstrate their existence unequivocally in most corneas. Their orientation, frequency and extent varies with pathological syndromes such as keratoconus, corneal opacity and edema, and as such could be useful as a clinical indicator, not just of keratoconus but also of other pathologies. Their diminution in number and width with age is expected as the cornea becomes more rigid with age^18^. Their detection in multiple mammalian species (mouse, macaque, human) indicates their conservation in evolution.

### Structure of striae

The literature states^5–7^ that striae are related to arrangement of collagen lamellae. Lamellae (which are 1–3 µm thick), are at the resolution limit of FFOCM and CM and lack contrast to be individually visible in OCT, FFOCM, or CM. Our assessment of cross-sections of corneal stroma with SHG, histology, immunofluorescence and SEM shows that the striae are caused by undulations in continuous lamellae rather than rupture or joining of different lamellae. P-SHG clearly demonstrates a change in orientation of the collagen fibrils at striae in transverse images with a quantifiable angle of around 70°. Striae are observed both with *ex vivo* technologies (FFOCM, SHG, histology and SEM) and *in vivo* technologies (OCT and CM) showing they are not linked to changes in the physiological conditions (i.e. absence of intraocular pressure, absence of metabolism flux or changes in the stromal hydration rate) related to isolation of cornea from the eyeball. CM, FFOCM and OCT show the striae as dark bands because at the position of the striae, the undulated lamellae reflect the light away from normal incidence, so that the back-reflected signal is absent in this zone and the striae appear dark. SHG also shows striae as dark because the SHG signal is lower for collagen fibrils tilted out of the focal plane. These undulations run through the corneal stroma from Descemet’s membrane toward Bowman’s layer. All technologies show that they depart from Descemet’s membrane. They appear to end in the mid or anterior stroma, or can reach Bowman’s layer. Furthermore, FFOCM, CM, SHG, and histology clearly demonstrate criss-crossing orientations of striae in the plane parallel to the ocular surface.

Stromal striae are rich in collagen VI and laminin. Collagen VI is a filamentous collagen that has been shown to play a role in the tensile strength and transparency of the stroma^19^. It is bound to the striated collagen fibrils by mediation of glycosaminoglycans or proteoglycans such as lumican^20^. In corneal stroma, keratan sulfate-containing proteoglycans include keratocan, lumican and mimecan which all belong to the small leucine-rich proteoglycan (SLRP) family. The corneal stroma also contains decorin which is a chondroitin sulfate-containing proteoglycan. Interestingly we found that stromal striae are rich in keratan-sulfate-containing proteoglycans (i.e., keratocan and lumican) but not in decorin. Lumican and keratocan have been shown to be involved in maintaining corneal shape and stromal transparency^21–24^.

### Functions of striae

Our experimental results on human donor and macaque cornea mounted in an artificial chamber, submitted to various pressure levels, show that striae are less visible with increasing posterior pressure. This effect does not depend on the stromal hydration level as the corneal central thickness was unchanged during experiments using CM and OCT imaging. In fact the stromal hydration rate is linearly linked to corneal central thickness^25,26^. Considering that under pressure, the undulations are erased, like a blown up balloon that loses its wrinkles, we can hypothesize that striae could protect collagen fibrils from damage or rupture induced by external mechanical shocks and subsequent increased intraocular pressure. The cornea is known to provide the structural stability required to protect the fragile intraocular components^27^. Of note, in physiological conditions, intraocular pressure dramatically increases during eye rubbing. Considering the cornea as a dome inserted in a rigid peripheral scleral rim with a posterior stress driven by intraocular pressure and the collagen fibril organization in the corneal stroma, a high level of intraocular pressure could result in corneal collagen fibril damage. In the corneal stroma, most of the collagen fibrils run in lamellae parallel to the corneal surface often communicating with those of adjacent layers by interchanging their fibrils. Conversely in sclera, collagen fibrils form bundles often giving off branches, and intertwining with one another^28^. Experimental data in human corneas have shown that the distribution of collagen fibrils within lamellae does not change at increasing IOP, while inter-lamellar re-organization enables evolution towards a more balanced distribution of orientation^29^. Experimental data in porcine eyes show 1 - the scleral modulus of elasticity to be 3 to 3.5 times that of the cornea (i.e., sclera is more rigid than cornea) and 2 - cornea to be more resistant than sclera to IOP changes within the physiological range^30^. The latter property could be explained at least partly by flattening of lamellae undulations at the level of striae as a response to increased IOP. This mechanical behavior appears similar to the uncrimping of collagen fibers in tendons as a response to traction^31,32^ or the action of sacrificial bonds in bone^33^. It is also associated with stable corneal radius of curvature with increased intraocular pressure which is important for maintenance of retinal image quality despite variations in intraocular pressure^30^. Having multiple orientations of striae in the plane parallel to the ocular surface is probably important in order to absorb the increase in posterior stress in all meridians of the corneal surface. Therefore we speculate that the first physiological function of striae could be to protect stromal ultrastructure and corneal shape from consequences of external mechanical shocks (eye rubbing) and subsequent increased intraocular pressure.

Cornea exhibits viscoelastic behavior which is clinically assessed by measurement of hysteresis^34,35^: the strain-stress curve of cornea is modified between the loading cycle and the unloading cycle. The anatomical structure supporting corneal elasticity is not yet fully established. On the one hand, cornea is devoid of elastin fibrils which are major features of elastic tissues. Elastin-free microfibril bundles are present during embryonic and early postnatal development and could be also found in adult corneas^36^. They could support corneal elasticity at least partly. On the other hand, the corneal stroma extracellular matrix is rich in proteoglycans which allow collagen fibrils to slide over each other without friction and collagen fibrils themselves can exhibit some elastic properties^37–39^.

We hypothesize that stromal striae could be associated with corneal viscoelasticity. This hypothesis is supported by two features: 1 – stromal lamellar undulations (striae) are erased when intraocular pressure (which represents a shearing force) is increased and recover when pressure is decreased, which corresponds to an elastic behavior 2 – this implies that parts of the long stromal collagen fibrils or lamellae slide over each other to adjust their position, which is consistent with presence of creep (a feature of viscous materials), 3 - shear wave velocity is locally modified at the level of striae. The elastic properties allow the corneal shape to be maintained and the viscous properties permit dissipation of energy. Determining the true shear wave velocity at the level of striae is still not possible because the stromal lamellae undulation width is smaller than the resolution of current shear wave technologies (150 µm for ultrafast ultrasonic imaging^40^, and 20 µm for OCT-SWE). However, OCT-SWE images showed that the striae have lower elasticity than surrounding stroma.

### Role of striae in keratoconus pathophysiology

*In vivo* corneal elasticity can be assessed with an elasticity coefficient derived from the Reichert Ocular Response Analyzer data^34,41^. Compared with normal cornea, this elasticity coefficient is lower in keratoconic corneas and corneas after laser *in situ* keratomileusis. The corneal hysteresis, which increases with corneal viscosity, is decreased in keratoconic corneas even at an early stage of the disease, showing keratoconic cornea are less viscous than normals^42,43^. This decrease in hysteresis is at least partly explained by decreased corneal thickness^44^. Keratoconic corneas feature localized thinning with focal reduction in elastic modulus (i.e., reduction in the meridian elastic modulus or in the shear modulus perpendicular to the corneal surface)^45^. Similarly, confocal Brillouin microscopy shows a lower shift in the anterior stroma in the keratoconus zone compared with both regions away from the apex of the cone and normal corneas, showing that mechanical loss is primarily concentrated within the area of the cone^46^. Of note, stromal striae are mainly found in the keratoconus zone. We previously demonstrated that presence of striae is associated with a high risk of acute progression of keratoconus characterized by Descemet’s membrane rupture (hydrops)^47^. All these considerations lead to consider striae as important features of keratoconus pathophysiology associated with local change in corneal viscoelasticity and lower hysteresis and lower stiffness.

If we consider that the increased number and change to vertical orientation of striae are associated with changes in corneal viscoelasticity and subsequent degraded biomechanical behavior, it is therefore interesting to detect and analyze them for keratoconus diagnosis. Detection and analysis of striae may also be useful in patient selection for refractive surgery procedures.

### Striae and glaucoma

Stromal striae could play a role in glaucoma pathophysiology which includes increased ocular rigidity^48^. Thin cornea is an important risk factor for progression of glaucoma^49^. Furthermore decreased corneal hysteresis may be strongly associated with glaucoma presence and risk of progression^50^. If the biomechanics of the cornea are unusual (indicated by changes in striae), then we may hypothesize that this may be another situation where glaucoma will be more likely.

## Conclusion

We have demonstrated the existence of a new structure in corneal stroma that was previously overlooked as a clinical indicator of only keratoconus. In fact, stromal striae were detected in 82% of 118 human corneas. Stromal striae appear to play a role in the biomechanical viscoelastic properties of the cornea, and as such they are important to the understanding of the behavior of the cornea in both physiological and clinical contexts.

## Methods

### Study design and ethics

This was a prospective observational case study. Informed consent was obtained from all patients. No modifications to French standards of treatment or follow-up were made. Institutional Review Board (IRB) approval was obtained from the Patient Protection Committee, Ile-de-France V (14944). The study was carried out according to the tenets of the Declaration of Helsinki and followed international ethical requirements for human tissues.

All animal manipulation was approved by the Quinze Vingts National Ophthalmology Hospital and regional review board (CPP Ile-de-France V), and was performed in accordance with the ARVO Statement for the Use of Animals in Ophthalmic and Vision Research.

### Human subjects and tissues

118 human corneas were assessed in total, of which 89 pathological, were grouped by syndrome (i.e.normal, keratoconus, edema (including Fuch’s dystrophy, bullous keratopathy) or opacity (including stromal scar and Schneider, Cogan, Reiss-Bücklers, granular and macular dystrophies)), as detailed in Table 1. Average overall age was 50 years (range 16–90), with average age per group of 55 years normal (of which 39 years healthy, 71 years donor), 37 years keratoconus, 64 years edema, 74 years opacity. Corneas that underwent deep anterior lamellar rather than penetrating keratoplasty were excluded from the *ex vivo* analysis since the posterior stroma was either not intact (lamellar dissociation induced by air injection) or not present in the *ex vivo* specimens. Within the keratoconus group of 45 pre-treatment corneas, 13 underwent keratoplasty and went on to be assessed with the *ex vivo* methods, while the remainder underwent treatment by cross-linking (10 corneas) and intrastromal ring segments (22 corneas), which we then assessed *in vivo* post-treatment, in order to determine the effect of these treatments on striae. No patient wore lenses during at least the 72 hours before examination. OCT-SWE was not successful on corneal buttons as the buttons could not be mounted in an anterior chamber, meaning that IOP could not be maintained or controlled. OCT-SWE was therefore only performed on normal donor or macaque cornea with intact scleral rims allowing anterior chamber mounting.

**Table 1:** Number of each type of cornea imaged with each technology.

Imaging technology	*In vivo*/*ex vivo*/*sections*	Normal human corneas	Keratoconus	Edema	Opacity	Intrastromal ring segment	Cross-linking	Normal macaque	Normal mouse
OCT	*In vivo*	13	45	31	13	22	10	0	0
CM (@ increasing IOP)	*In vivo (ex vivo)*	15(6)	45	31	13	22	10	2	0
FFOCT	*Ex vivo*	14	13	15	13	1	0	6	10
OCT-SWE	*Ex vivo*	4	0	0	0	0	0	5	0
SHG (@ increasing IOP)	*Sections*	2(6)	5	0	0	0	0	2	0
Histology (immuno)	*Sections*	10(3)	12(3)	6(1)	5	0	0	2(3)	0
SEM	*Sections*	1	0	0	0	0	0	0	0

Donor corneas were obtained from the tissue bank of the Etablissement Français du Sang, Ile-de-France (Paris, France) after they had been rejected for transplantation due to low endothelial cell count. The donor corneas were preserved in CorneaMax (EuroBio, France) medium for a maximum of 35 days at 31 °C, in accordance with European Eye Banking regulations. They were then placed in CorneaJet (EuroBio, France) medium containing Dextran for deturgescence 48 hours prior to imaging, and transferred to the Quinze-Vingts National Ophthalmology Hospital (Paris, France). Pathological corneal buttons were obtained during keratoplasty procedures performed on patients at the Quinze-Vingts National Ophthalmology Hospital. They were collected from the operating room at the time of the keratoplasty and conserved in CorneaJet (EuroBio, France) medium during imaging, and then fixed in 4% formaldehyde (FA) before transfer to the pathology laboratory for histological processing.

### Corneal hydration

The effects of corneal hydration were explored by imaging a human donor cornea using SD-OCT and CM while mounted in an anterior chamber at various pressure levels (0 to 60 mmHg, in 10 mmHg steps), perfused with organ culture deswelling medium (CorneaJet, Eurobio, Les Ulis, France) for 3 days (Fig. sup 1.4). SD-OCT and CM imaging were performed at 4 hydration points corresponding to advanced stromal edema (central corneal thickness (CTT) 900 µm), mild stromal edema (CCT 600 µm), normal stromal hydration (CCT 500 µm), and stromal dehydration (CCT 300 µm).

We sought to avoid or minimize edema throughout our study by 1) imaging striae *in vivo* using SD-OCT and CM, where corneal hydration was at a normal physiological level; 2) in all *ex vivo* imaging, we imaged pathological corneal buttons and macaque tissues as soon as possible after extraction from the eye, and conserved them in CorneaJet (Eurobio, Les Ulis, France) deturgescence transport medium; 3) we rejected any donor cornea of non physiological thickness after deturgescence.

### Animal tissues

Ten macaque ocular globes were obtained from a partner research facility and transported to the Vision Institute in CO_2_-free neurobasal-A medium (Gibco, Life Technologies, UK). Ten mouse ocular globes were obtained from the Vision Institute animal facility. Anterior segments were dissected from the globes, then lens and iris were removed to retain only the corneas which were stored in cornea transport medium (CorneaJet, Eurobio, France) or fixed before imaging. Imaging performed on these corneas is detailed in Table 1.

### *In vivo* imaging

The day before keratoplasty, patients were assessed *in vivo* with CM (Heidelberg Retina Tomograph III; Heidelberg Engineering GmbH, Heidelberg, Germany) and OCT (RTVue-100^©^, Optovue Inc, Fremont, CA). Normal corneas from healthy subjects were assessed *in vivo* with the same techniques. We acquired series of en face CM images (between 600 and 1600 per patient), at the conus for keratoconus and at the center for normals and other pathologies, moving down through the depth of the cornea, along with 6-mm wide OCT scans. Axial × lateral resolution of CM was 7.6 µm × 1 µm^51^, and 5 µm × 8 µm for OCT^52^.

### *Ex vivo* imaging

The FFOCM system used (LightCTScanner, LLTech, France) has been described previously^11^. Image stacks at the conus for keratoconus and at the center for normals and other pathologies were acquired on each button covering the entire corneal thickness, with an image acquired every micron in depth. Resolution in xyz planes is 1.5 µm × 1.5 µm × 1 µm. 3D image stacks could be examined in en face and cross-sectional views using the multiplanar reconstruction (MPR) software provided with the system. Further information on the technology is available in supplementary material section 2.1.

For histology, following FFOCM assessment, corneas were fixed (4% FA) and histological processing was carried out in the histopathology laboratory. Hematoxylin and eosin stain (HES) and periodic acid–Schiff (PAS) stains were performed on paraffin embedded material as these are routine stains for corneal pathology.

OCT-SWE was performed using a custom-developed setup that has been described previously^53^. Controlled shear waves were induced within corneas by a piezoelectric actuator placed at the sample surface and tracked using phase-sensitive spectral-domain OCT. With this custom setup, a lateral resolution of 9.5 μm and an axial resolution of 9.7 μm is achieved with a sensitivity on axial displacement of about 10 nm over a field of view of about 2.5 × 1.5 mm. Further information on the technology is available in supplementary material section 2.2. Corneas were mounted in an artificial chamber, and data acquired at pressure steps of 5 mmHg, from 5 mmHg to 60 mmHg. For comparison with the OCT-SWE results with regards to influence of IOP, these corneas mounted in an artificial chamber were also imaged with CM in the same sequence of pressure steps.

SHG microscopy was performed using a custom-developed laser scanning upright multiphoton microscope as previously described^17^. Images were acquired either with circularly polarized excitation or with a set of linear polarizations with different orientations to achieve polarization-resolved SHG microscopy and map the directions of collagen fibrils in the focal plane. Axial × lateral resolution was 1.8 µm × 0.4 µm. Further information on the technology is available in supplementary material section 2.3. Moreover, SHG data from a previous study were processed again to look for striae in relation to pressure; in this study, normal human corneas (n = 6) were mounted in a custom-built artificial chamber and images acquired at IOP steps of 12 mmHg, from 0 to 48 mmHg^25^.

For collagen labeling, paraffin embedded samples were incubated for 1 hour in PBS containing 0.3% Triton × 100 and 0.2% gelatin (PBSGT) to block nonspecific binding. Cells were incubated for 1 night at 4 °C with specific primary antibodies (Col IV (DAKO, 1/100), Col V (Abcam, 1/100), Col VI (Sigma, 1/300), lumican (Sigma, 1/200), keratocan (Santa Cruz Biotechnology, 1/200) and decorin (Santa Cruz Biotechnology, 1/100) diluted in PBST. After washing, samples were reacted for 1 hour at room temperature with the appropriate secondary antibodies diluted in PBST. The following secondary antibodies were used: labeled goat anti-mouse IgG antibody (Alexa Fluor 488, 1:500; Sigma) and labeled goat anti-rabbit IgG antibody (Alexa Fluor 594, 1:500; Sigma). Nuclei were counterstained with Dapi (1:1000; Sigma). Samples were washed 3 times, 5 min each with PBST. Stained samples were observed with a laser scanning confocal microscope.

Scanning electron microscopy (SEM) was performed on eye bank corneas (provided by the Banque Française des Yeux, Paris, France) in the context of an earlier study^54^. After dissection with a scalpel, the corneas were fixed in 2.5% glutaraldehyde cacodylate buffer (0.1 M, pH 7.4). Specimens were additionally fixed in 1% osmium tetroxyde in cacodylate buffer (0.2 M, pH 7.4) and subjected to successive dehydration in graduated ethanol solution (50%, 70%, 95%, and 100%) then in propylene oxide. The SEM procedure was performed in a specialized laboratory (Service de Microscopie Électronique, Institut de Biologie Intégrative IFR 83, Université Pierre et Marie Curie – Paris VI, Paris, France) after being dehydrated and coated with a thin gold layer.

### Image analysis

CM, OCT, FFOCM and histology images were analyzed by expert observers (one corneal surgeon, one imaging expert, one orthoptist, one pathologist). They made a qualitative description, and a quantitative tally, of the number, dimensions, and orientation of striae according to pathology. ImageJ software (NIH, Bethesda, USA) was used to display images and perform quantitative measurements. SHG, OCT-SWE, collagen labeling and EM data was processed and presented by those authors expert in each field, and results analyzed by the author group. Further information on the quantitative analysis of SHG data is available in supplementary material section 2.3.

### Statistical analysis

Comparison of groups was made with one-way ANOVA with appropriate post-hoc tests or Wilcoxon sign rank test for continuous variables. The Spearman correlation coefficient was used to assess correlation between continuous variables. For qualitative variables, comparison of groups was made with the chi-square test.

### Data availability

The datasets generated during and/or analyzed during the current study are available from the corresponding author on reasonable request.

## Electronic supplementary material

Supplementary material
