# Supplementary material for: Stromal striae: a new insight into corneal physiology and mechanics
Source: Sci Rep. 2017 Oct 19;7:13584. doi: 10.1038/s41598-017-13194-6 (PMC5648881; doi:10.1038/s41598-017-13194-6)
Supplement: Supplementary file 1 — Supplementary material [file 41598_2017_13194_MOESM1_ESM.pdf]

Stromal striae: a new insight into corneal physiology and mechanics

Kate Grieve[1, 2], Djida Ghoubay[1, 2], Cristina Georgeon[1], Gael Latour[3], Amir Nahas[4], Karsten Plamann[5], Caroline Crotti[5], Romain Bocheux[4], Marie Borderie[1], Thu-Mai Nguyen[4], Felipe Andreiuolo[1], Marie-Claire Schanne-Klein[6], Vincent Borderie[1, 2]

[1]CHNO des Quinze Vingts, [2]Institut de la Vision, [3]Laboratoire Imagerie et Modélisation en Neurobiologie et Cancérologie, Univ. Paris-Sud, CNRS, Université Paris-Saclay, Orsay, France, [4]Institut Langevin, [5]ENSTA ParisTech, Palaiseau, France, [6]Laboratoire d'Optique et Biosciences, Ecole polytechnique, CNRS, INSERM U1182, Université Paris-Saclay, Palaiseau, France.

Supplementary material

Sup 1   Supplementary Results

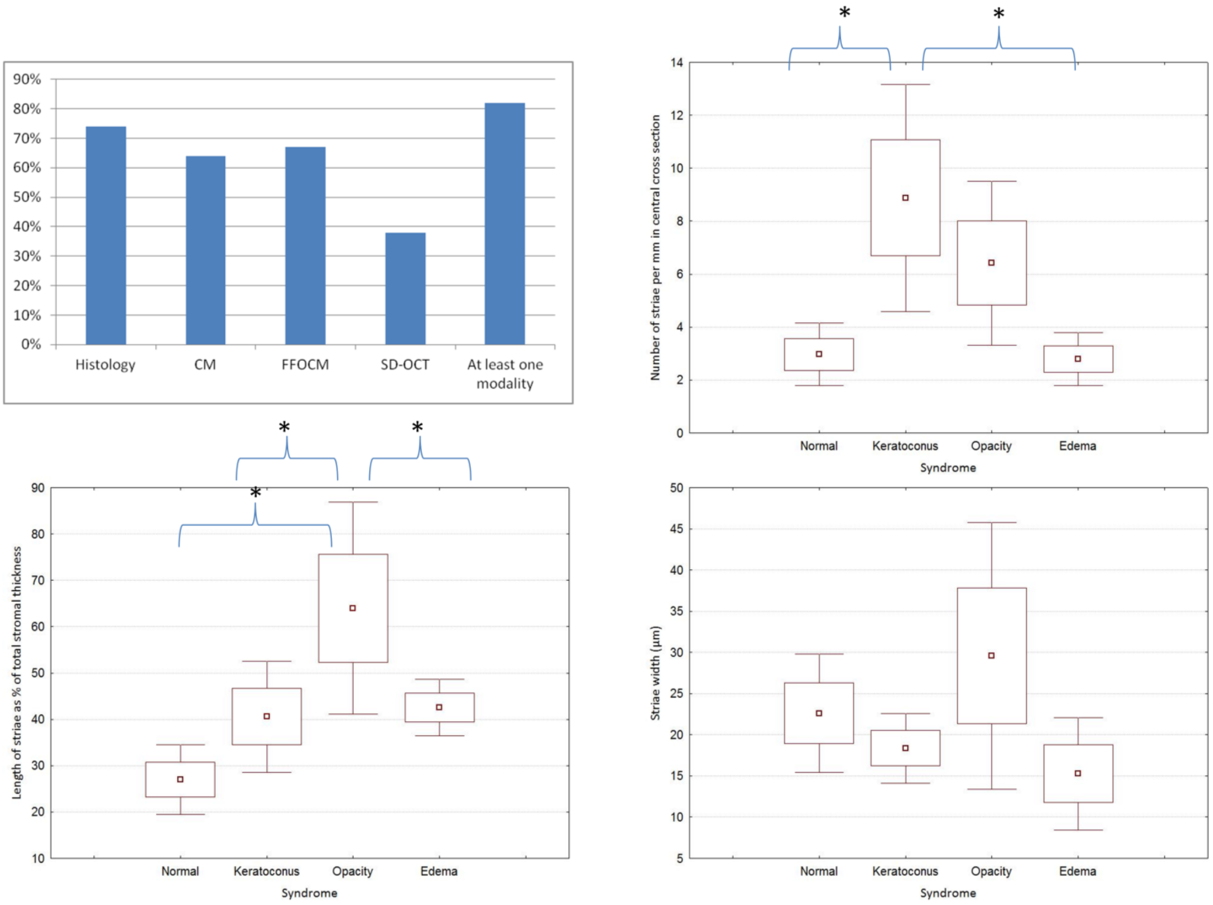

Fig sup 1.1: Top left: histogram of percentage of corneas in which striae detected, per modality. Top right and bottom: graphs showing significant differences in striae parameters.Top right: number of striae per mm for each syndrome; bottom left: length of striae as a percentage of total corneal thickness for each syndrome; bottom right: width of striae in microns for each syndrome. *indicates statistical significance (p<0.01). The central dot shows the mean, the boxes show the standard error on the mean and the error bars show the confidence interval (=1.96 x standard error on the mean).

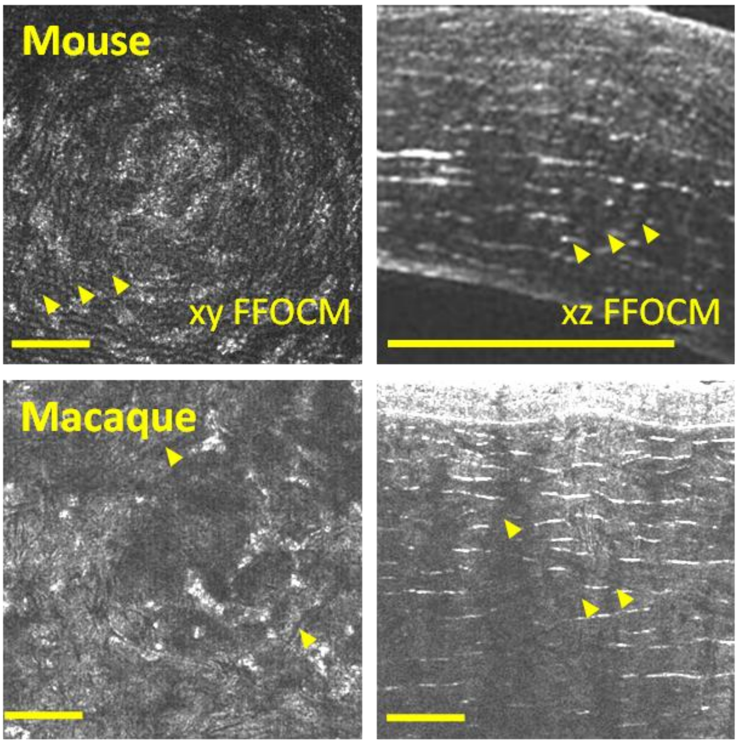

Fig sup 1.2: Striae in other mammalian species (mouse: top, macaque: bottom), as seen in en face xy (left) and cross-sectional xz (right) views in FFOCM. Scale bars show 100µm.

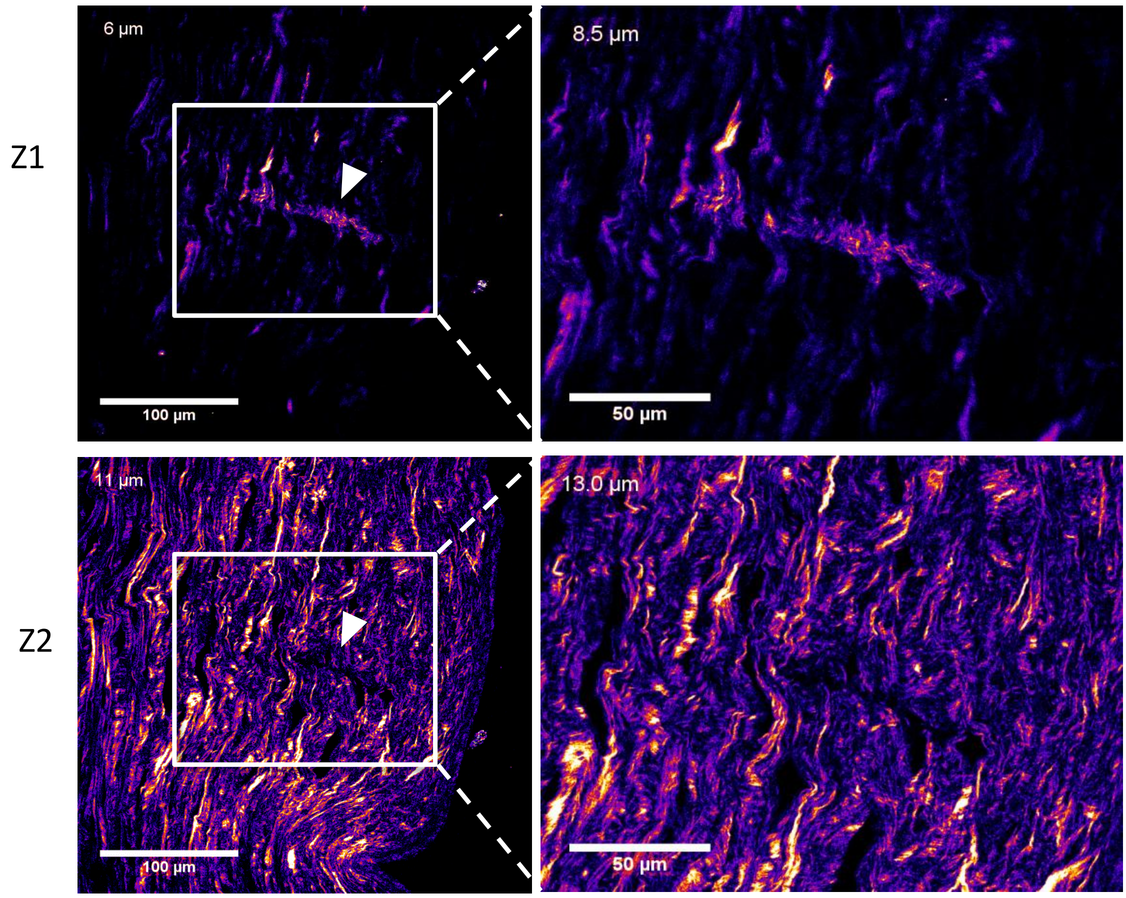

Fig sup 1.3: SHG imaging of folding artifact. SHG images at different depths within the histological section shows the 3D shape of an artefactual fold. This fold exhibits SHG signal at a depth different from the rest of the histological section.

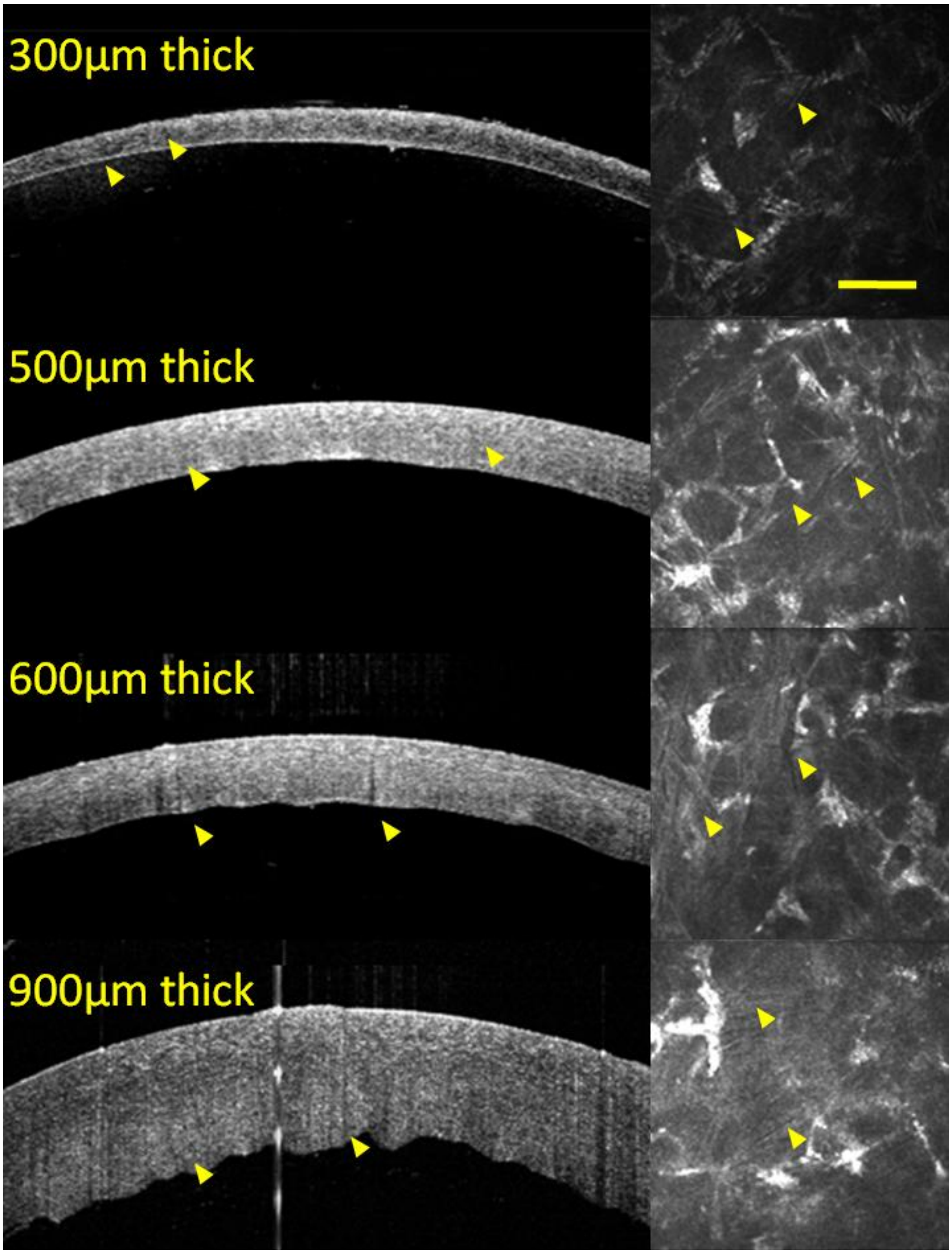

Fig sup 1.4: A normal human donor cornea was mounted in an artificial chamber and perfused with organ culture deswelling medium for 3 days. SD-OCT and CM imaging were performed at 4 hydration points corresponding to advanced stromal edema (central corneal thickness (CTT) 900 µm), mild stromal edema (CCT 600 µm), normal stromal hydration (CCT 500 µm), and stromal dehydration (CCT 300 µm). Stromal striae were clearly observed in the anterior, mid and posterior stroma at all corneal thicknesses and all pressure levels (0mmHg to 60mmHg) with CM. SD-OCT showed that striae are more visible when stromal hydration increases. Figure panels show CM and SD-OCT at 20mmHg; arrowheads indicate striae; scale bar 100µm.

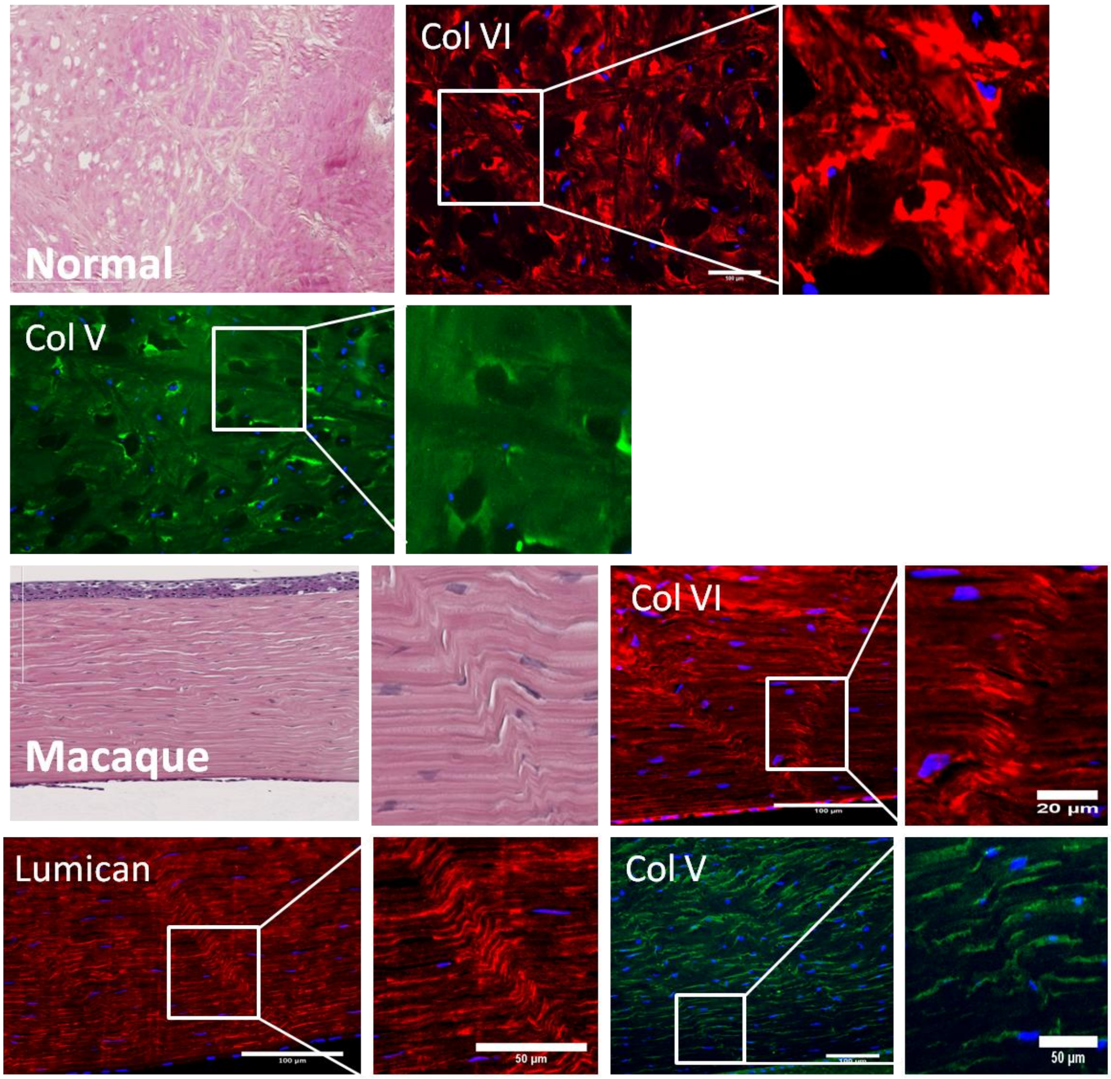

Fig sup 1.5: Immunohistology and histology en face views of human normal and keratoconus cornea, and views of normal macaque cornea, confirming the presence of collagen VI and lumican within striae. Scale bars show 100µm.

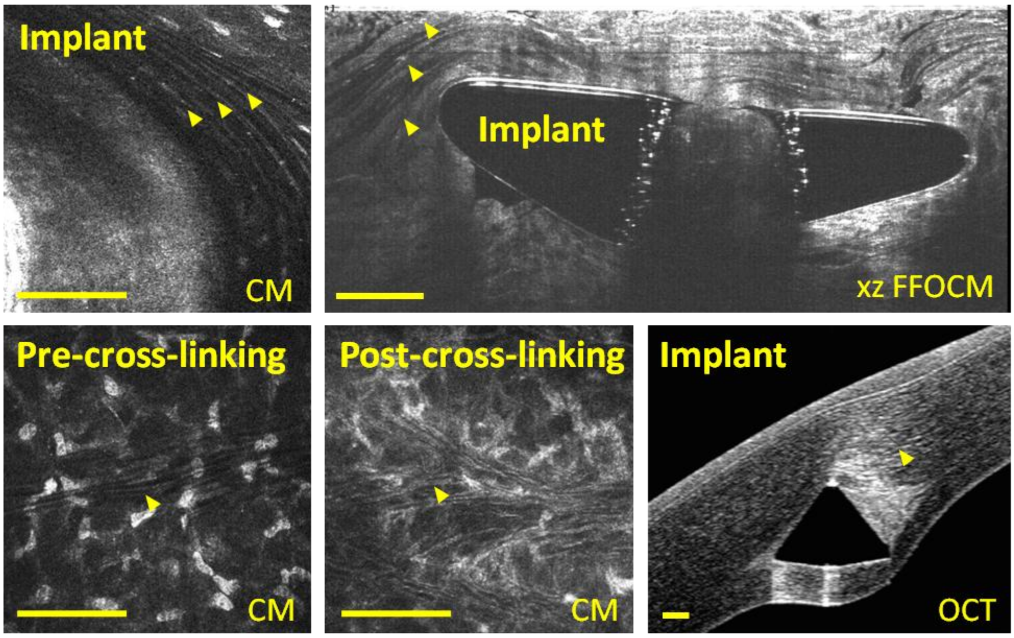

Fig sup 1.6: Striae in treated keratoconus corneas. Surrounding an intrastromal ring segment, and before and after cross-linking. Viewed with (clockwise) confocal microscopy (CM), cross-sectional (xz) FFOCM and OCT views. Striae were observed surrounding the intrastromal ring segment in orientations that followed the annular form. Bottom left and center: pre and post cross-linking, where striae contrast remains constant while surrounding stromal hyperreflectivity increases. Scale bars show 100µm.

Sup 2 Supplementary methods

Sup 2.1 FFOCM imaging method

The experimental arrangement of FF-OCT (Figure sup 2.1) is based on a configuration that is referred to as a Linnik interferometer. A halogen lamp is used as a spatially incoherent source to illuminate the whole field of an immersion microscope objective. The signal is extracted from the background of incoherent backscattered light using a phase-shifting method implemented in custom-designed software. This study was performed on a commercial FF-OCT device (LightCT, LLTech, Paris, France). This FF-OCT microscope is housed in a compact setup that is about the size of a standard optical microscope.

FF-OCT provides the highest OCT 3D resolution of 1x1x1 μm^3 (XYZ) on unprepared label-free tissue samples over a wide field of view that allows digital zooming down to the cellular level. In contrast to conventional OCT, it produces en face images in the native field view, similar to confocal microscopy

images (i.e. with similar lateral yet superior axial resolution), and the cross-sectional views are then reconstructed from the 3D data stack.

It combines attributes of confocal and OCT techniques to provide both high-resolution en face and cross-sectional views in a single instrument. FF-OCT image acquisition and processing time is under five minutes for an 800 µm x 800 µm x 500 µm depth stack in the cornea.

FF-OCT image contrast is generated by the same principles as in conventional OCT, i.e. it shows differences in light backscattering between the different tissue components, or in other words shows differences in refractive index. The most significant difference between FF-OCT and conventional OCT is the higher resolution achieved. The axial resolution improvement is thanks to the use of a white light source illuminating the whole field as compared to a laser source scanning the field point by point. This in turn means that microscope objectives may be used to improve transverse resolution (possible due to the reduced depth of field in FF-OCT, and hence implying a trade-off of reduced penetration depth), and image capture of the whole field can be performed in a single shot by a complementary metal-oxide semiconductor (CMOS) camera as opposed to a single pixel detector. The resulting native image orientation is therefore en face in FF-OCT versus cross-sectional in conventional OCT. These technical factors result in resolution of 1 µm 3D versus around 5–15 µm 3D in conventional OCT. Penetration depth in FF-OCT is around 1mm in cornea versus over 1mm in most tissues with conventional OCT.

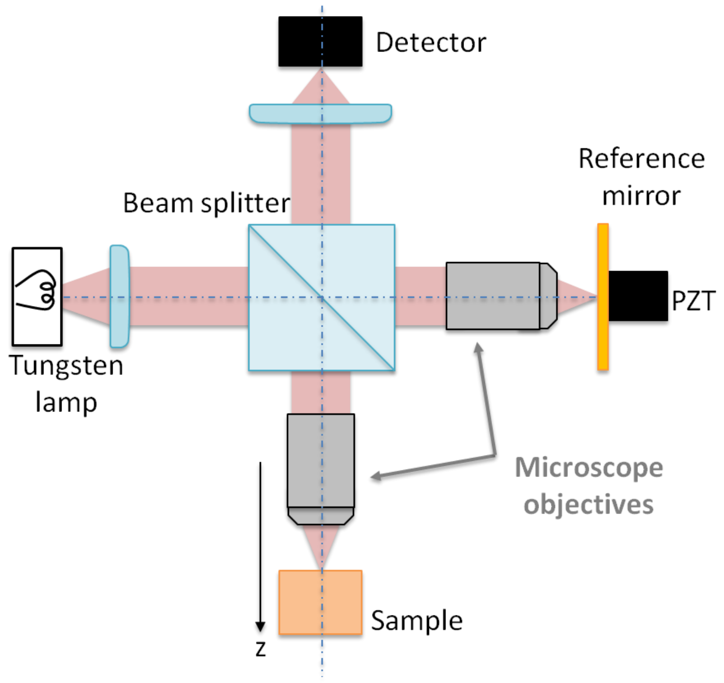

Fig sup 2.1 Experimental setup of the FF-OCT device. PZT is a piezoelectric stage.

Sup 2.2 OCT-SWE imaging method

Our fiber-based OCT setup uses a broadband light source from a superluminescent diode (1050-nm central wavelength, 50-nm bandwidth, 25-mW maximal output power; MWTechnologies, Moreira da Maia, Portugal). The output power is split between a reference arm (20%) and a sample arm (80%). The interference signal occurring between the two arms is directed through a circulator towards a high-speed spectrometer operating at 60-kHz A-line rate (2048 pixels, 1000-1100 nm spectral range, 0.2-nm spectral resolution – Wasatch Photonics, Durham, NC, USA). In the sample arm, two galvo-mirrors scan the light beam laterally (along x and y) across the sample. All devices are synchronized by a computer running under a custom-developed Matlab© (MathWorks, Natick, MA, USA) software.

The setup can be operated in two scanning modes. In the first mode (C-mode), volumetric static morphologic images of the sample are acquired by scanning the light beam laterally across the sample. The acquisition time for a volumetric image is 1.4 sec for a field-of-view of 3.8 x 3.8 x 2 mm^3 and a voxel size of 14 x 14 x 5 μm^3 (x,y,z). In the second mode (M-mode), dynamic displacements are sampled in time at a fast rate by recording successive acquisitions at one given location.

A piezoelectric actuator (Thorlabs AE0505D18F – Newton, NJ, USA) is placed in contact with the sample surface to generate a propagating shear wave, i.e. axial displacements propagating laterally. The actuator is driven by a function generator (Agilent 33220A) emitting a 6-kHz, 0.5-ms long, 10V peak-to-peak amplitude, sine wave. In order to follow the propagation of shear waves across the sample, several M-modes are recorded at different lateral locations while the piezoelectric excitation is repeated, resulting in a stroboscopic tracking of the shear wave at 60-kHz equivalent frame rate. In this study, we limited the recording of the displacement to a 2D cross-section, with a total acquisition time of 1.2 sec for a field-of-view of 3.8 x 2 mm^2 and a pixel size of 14µm x 5µm (transverse x axial).

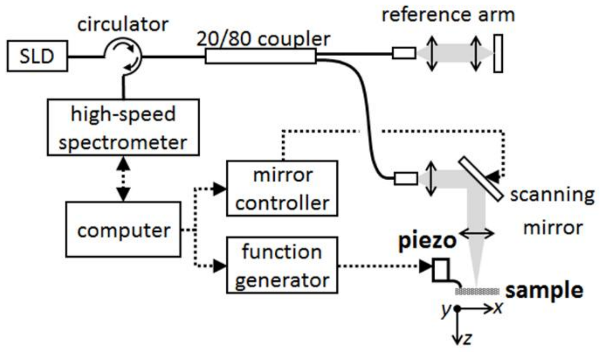

Fig sup 2.2 – Fiber-based spectral-domain OCT setup for dynamic elastography. The light source is a broadband superluminescent diode (SLD, 1050-nm central wavelength, 100-nm spectral bandwidth). The detector is a high-speed spectrometer (76-kHz maximal line rate). In the reference arm, light is

focused on a fixed gold-coated mirror. In the sample arm, two galvo-mirrors scan the sample transversally (along x and y). Micrometric displacements are generated within the sample using a piezoelectric actuator positioned at the sample surface. All devices are synchronized by a computer through a Matlab© script.

Sup 2.3 SHG and P-SHG imaging method and processing

SHG microscopy was performed using a custom-built laser scanning multiphoton microscope as previously described[17] (Fig sup 2.3 a). Excitation was provided by a femtosecond Titanium-Sapphire laser (Mai-Tai, Spectra-Physics) tuned at 860 nm. We used either circular polarization in order to image collagen fibrils independently of their orientation in the image plane (SHG), or linear polarization with different orientations in order to perform polarization-resolved measurements (P-SHG). Transverse sections were imaged with a 25x 1.05 NA objective (XLPLN 25X W MP2, Zeiss). Power at the sample was 7 to 12 mW with 5 µs pixel dwell time and 0.4 µm pixel size. SHG signals were detected in the trans-direction using appropriate spectral filters (FF01-427/10 interferential filter and 2 FF01-680SP rejection filters, Semrock). P-SHG images were acquired at 19 different excitation angles every 10° between 0° and 180°. The P-SHG data were processed as previously described[17] using Fast Fourier Transform of the polarization data, which provided the main orientation of the collagen fibrils in the image plane (Fig sup 2.3 b).

SHG en face images of *ex vivo* corneas at increasing pressure were reprocessed from a previous study[25]. Briefly, SHG-image stacks were acquired every 50 µm along the full depth of corneas using circular excitation and epi-detection (see Fig sup 2.3 c). The same region of the cornea was imaged at all pressures thanks to fluorescent beads deposited at the corneal surface. The contrast of striae was assessed quantitatively by plotting transverse profiles of the SHG signal across striae, fitting with a negative Gaussian peak and computing the contrast as the normalized difference between the maximum signal (outside striae) and the minimum signal (inside striae): $C = \frac{Max-Min}{Max+Min}$ (see Fig sup 2.3 d). The values obtained from 3 profiles were averaged for every en face SHG images, and the ratio of the contrast at maximum pressure (50 mmHg) to the one at physiological pressure (12 mmHg) was computed in the middle and in the posterior stroma of 6 corneas.

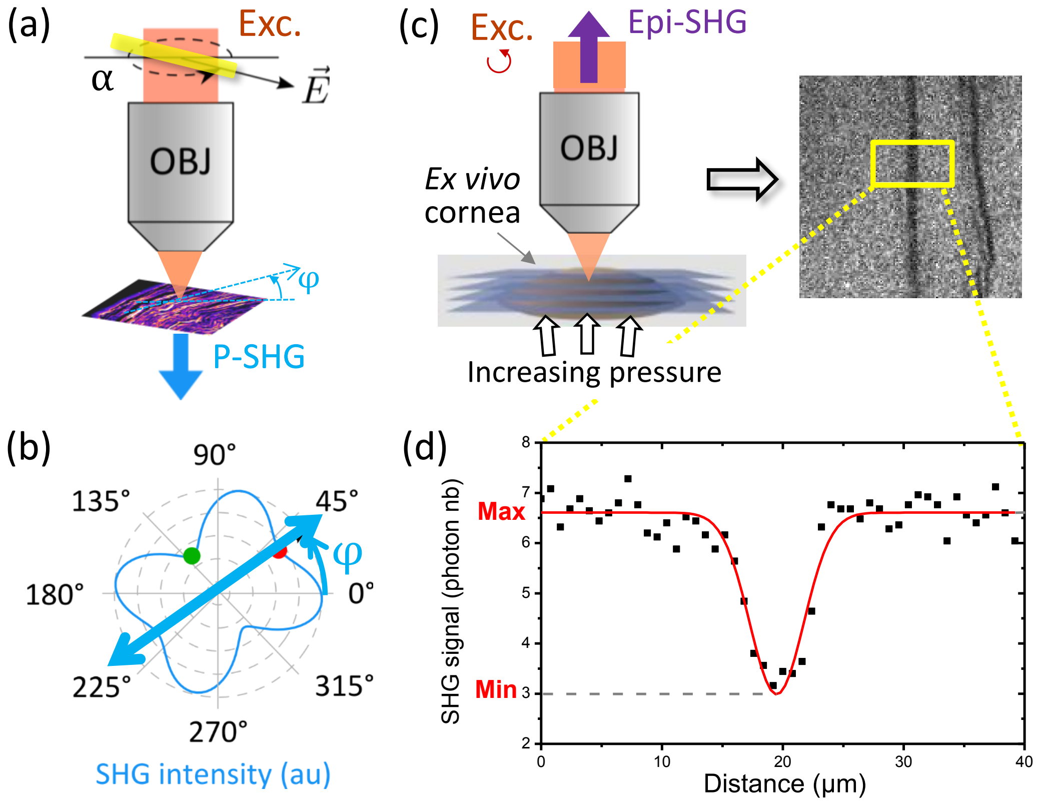

Fig sup 2.3: SHG and P-SHG setups and data processing. (a) P-SHG imaging of a transverse corneal section provides (b) an angular diagram of the SHG signal for varying orientations of the excitation polarization. The direction of the collagen fibrils in the section plane is given by the main direction φ of the diagram. (c) SHG epi-imaging of *ex vivo* cornea at increasing pressure using circular incident polarization provides en face images of striae. (d) SHG signal profile along the yellow rectangle and Gaussian fitting of the striae. The contrast is defined as $C = \frac{Max-Min}{Max+Min}$ and averaged over 3 profiles in every en face SHG image.
